# Receptor–Receptor Interactions in Multiple 5-HT1A Heteroreceptor Complexes in Raphe-Hippocampal 5-HT Transmission and Their Relevance for Depression and Its Treatment

**DOI:** 10.3390/molecules23061341

**Published:** 2018-06-03

**Authors:** Dasiel O. Borroto-Escuela, Manuel Narváez, Patrizia Ambrogini, Luca Ferraro, Ismel Brito, Wilber Romero-Fernandez, Yuniesky Andrade-Talavera, Antonio Flores-Burgess, Carmelo Millon, Belen Gago, Jose Angel Narvaez, Yuji Odagaki, Miklos Palkovits, Zaida Diaz-Cabiale, Kjell Fuxe

**Affiliations:** 1 Department of Neuroscience, Karolinska Institutet; Retzius väg 8, 17177 Stockholm, Sweden; dasiel.borroto.escuela@ki.se (D.O.B.-E.); ismebr@gmail.com (I.B.); 2 Department of Biomolecular Sciences, University of Urbino Carlo Bo, 61029 Urbino, Italy; patrizia.ambrogini@uniurb.it; 3 Observatorio Cubano de Neurociencias, Grupo Bohío-Estudio, Zaya 50, 62100 Yaguajay, Cuba; 4 Instituto de Investigación Biomédica de Málaga, Facultad de Medicina, Universidad de Málaga, 29071 Málaga, Spain; mnarvaez@uma.es (M.N.); afburgess@uma.es (A.F.-B.); carmelomp@uma.es (C.M.); bgago@uma.es (B.G.); bueno@uma.es (J.A.N.); zaida@uma.es (Z.D.-C.); 5 Department of Life Sciences and Biotechnology (SVEB), University of Ferrara, 44121 Ferrara, Italy; frl@unife.it; 6 Department of Cell and Molecular Biology, Uppsala University,75105 Uppsala, Sweden; wromfdez@icm.uu.se; 7 Department of Neurobiology, Care Sciences and Society, Center for Alzheimer Research, Neuronal Oscillations Lab, Karolinska Institutet, 171 77 Stockholm, Sweden; yuniesky.andrade-talavera@ki.se; 8 Department of Psychiatry, Saitama Medical University, 3388570 Saitama, Japan; odagaki@saitama-med.ac.jp; 9 Department of Anatomy, Histology and Embryology. Faculty of Medicine. Semmelweis University, H-1094 Budapest, Hungary; palkovits.miklos@med.semmelweis-univ.hu

**Keywords:** heteroreceptor complexes, G protein-coupled receptors, oligomerization, receptor-receptor interactions, serotonin 5-HT1A receptor, depression, galanin, receptor tyrosine kinase, fibroblast growth factor receptor

## Abstract

Due to the binding to a number of proteins to the receptor protomers in receptor heteromers in the brain, the term “heteroreceptor complexes” was introduced. A number of serotonin 5-HT1A heteroreceptor complexes were recently found to be linked to the ascending 5-HT pathways known to have a significant role in depression. The 5-HT1A–FGFR1 heteroreceptor complexes were involved in synergistically enhancing neuroplasticity in the hippocampus and in the dorsal raphe 5-HT nerve cells. The 5-HT1A protomer significantly increased FGFR1 protomer signaling in wild-type rats. Disturbances in the 5-HT1A–FGFR1 heteroreceptor complexes in the raphe-hippocampal 5-HT system were found in a genetic rat model of depression (Flinders sensitive line (FSL) rats). Deficits in FSL rats were observed in the ability of combined FGFR1 and 5-HT1A agonist cotreatment to produce antidepressant-like effects. It may in part reflect a failure of FGFR1 treatment to uncouple the 5-HT1A postjunctional receptors and autoreceptors from the hippocampal and dorsal raphe GIRK channels, respectively. This may result in maintained inhibition of hippocampal pyramidal nerve cell and dorsal raphe 5-HT nerve cell firing. Also, 5-HT1A–5-HT2A isoreceptor complexes were recently demonstrated to exist in the hippocampus and limbic cortex. They may play a role in depression through an ability of 5-HT2A protomer signaling to inhibit the 5-HT1A protomer recognition and signaling. Finally, galanin (1–15) was reported to enhance the antidepressant effects of fluoxetine through the putative formation of GalR1–GalR2–5-HT1A heteroreceptor complexes. Taken together, these novel 5-HT1A receptor complexes offer new targets for treatment of depression.

## 1. Introduction

In membrane preparations of various central nervous system (CNS) regions, it was found in the 1980s that neuropeptides such as CCK8 and neurotensin could modulate the binding characteristics via their receptors, especially the affinity of the monoamine receptors in a receptor subtype-specific way [1,2,3]. In 1993, the concept was introduced that such receptor–receptor interactions took place in heterodimers [4]. In 2010, the role of higher-order heteromers was also underlined as being centers of integration, as demonstrated in cellular models [5]. In tissues, it is better to speak of heteroreceptor complexes, because little is known of their composition and stoichiometry and of the participation of adaptor proteins [6,7]. Thus, a number of proteins can bind to the receptor protomers, such as GPCR-interacting proteins (adaptor proteins), which can vary from one brain region to another as well as the stoichiometry of the participating receptors. Also, other receptors can be added to the heteroreceptor complexes in a dynamic way [8]. Furthermore, ion channels and transmitter transporters can also participate in such heteroreceptor complexes, increasing their signaling panorama. As a result of such changes, their allosteric receptor–receptor interactions can become altered. The allosteric receptor–receptor interactions in heteroreceptor complexes give diversity and bias to the receptor protomers due to conformational changes in discrete receptor domains altering receptor protomer function and pharmacology [8,9].

Another term used is “isoreceptor complex”. In contrast to the heteroreceptor complex, the different receptors in the isoreceptor complex always bind the same transmitter, for example, serotonin (5-HT). Thus, the 5-HT1A–5-HT2A receptor complex represents an isoreceptor complex, while the 5-HT1A–FGFR1 receptor complex represents a heteroreceptor complex.

The overall architecture of the global GPCR heterodimer network [10] shows a scale-free topology, because most protomers participate only in a couple of interactions. However, a few have more than ten connections (heterodimerization) to other GPCR protomers such as D2R and 5-HT1A receptors, in addition to direct interactions with receptor tyrosine kinase (RTK) [6,11,12,13,14,15,16] and ligand-gated ion channel receptors [17,18,19].

Serotonin receptor mechanisms play a major role in the development of depression and its treatment [20]. In 1967, the 5-HT uptake mechanism was found in the plasma membrane at the soma, axon, and terminal level of the central 5-HT neurons [21]. In 1968, Carlsson et al. reported that imipramine can block the 5-HT uptake mechanism, which led to the search for selective serotonin reuptake inhibitors (SSRIs) in the treatment of depression [22]. Postjunctional 5-HT1A receptors are today currently in the center of interest among the many 5-HT isoreceptors identified and regarded to be involved in the antidepressant actions of SSRIs [23,24,25,26].

Basic neurobiological research as well as clinical studies on SSRIs have established that disturbances in the ascending 5-HT neuron systems and their collateral networks to the forebrain, as well as their many 5-HT receptor subtypes, contribute to the etiology of depression and are targets for its treatment [14,23,26,27,28,29,30,31]. The therapeutic action of serotonin antidepressant drugs is of proven effectiveness, but the mechanisms underlying their effect are still unclear. There are many 5-HT receptor subtypes involved and some need to be blocked (e.g., 5-HT2AR, 5-HT3R, and 5-HT7R), while others need to be activated (e.g., postjunctional 5-HT1AR and 5-HT4R) [14,29]. Therefore, 5-HT subtype-selective antagonists or agonists can be used, inter alia, to enhance the antidepressant actions of SSRIs [29,32]. These state-of-the-art developments are in line with the hypothesis that the development of depression can involve an imbalance of the activity between different types of 5-HT isoreceptors [33,34]. Multi-targeting drugs, such as vortioxetine, with serotonin transporter-blocking properties together with a high affinity for a number of 5-HT isoreceptors are currently being tested for their potential to treat depressive disorders [26,35]. However, the 5-HT1AR remains in the center of interest [26,35,36].

**Figure 1:**
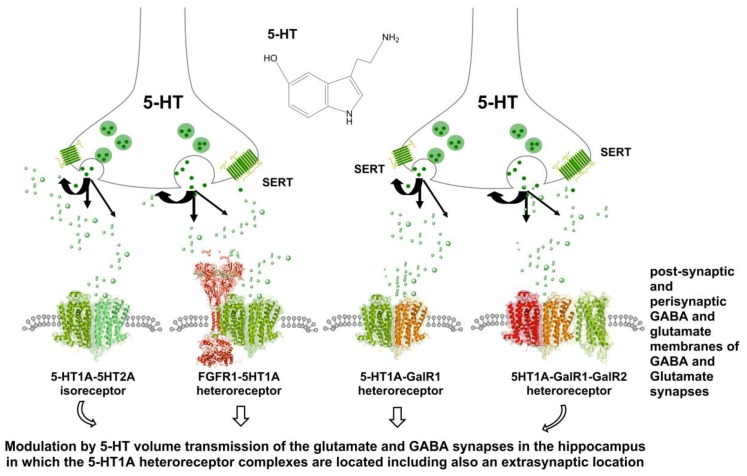
Illustration of how serotonin volume transmission can reach the 5-HT1A–FGFR1, 5-HT1A–5-HT2A, GalR1–5-HT1A, and GalR1–GalR2–5-HT1A heteroreceptor complexes. The ligand for the GalR1–GalR2 heterodimer is the galanin fragment Gal (1–15), while galanin (1–19) is the ligand for galanin receptor monomers and homomers. The 5-HT1A heteroreceptor complexes may mainly have an extrasynaptic location, but also a synaptic location. They are proposed to modulate synaptic glutamate transmission in pyramidal neurons of the hippocampus and synaptic GABA transmission in inhibitory GABA interneurons of the hippocampus. The differential role of these hippocampal 5-HT1A heteroreceptor complexes in modulating the hippocampal networks remain to be characterized.

It is known from our work that 5-HT1AR forms heteromers with many other receptors in neuronal membranes of the brain linked to the ascending 5-HT neurons [6,11,13,34,37,38,39,40,41,42,43,44]. They are of relevance for depression and its treatment. In this focused review, we will present an update of the work on the brain 5-HT1A–FGFR1 heteroreceptor complexes [6,11,13,14,39,40] and the demonstration of a novel 5-HT1A receptor complex: the brain 5-HT1A–5-HT2A isoreceptor complex [34]. The complex yet exciting world of brain GalR–5-HT1A heteroreceptor complexes will also be briefly discussed [37,42,43,45] (Figure 1).

## 2. FGFR1–5-HT1A Heteroreceptor Complexes

These complexes have been observed in the hippocampus and in the dorsal raphe 5-HT neurons [6,13,40,46] using the in situ proximity ligation assay (in situ PLA) [46,47,48] (Figure 1). In the dorsal raphe, the 5-HT1A receptor functions as an autoreceptor [25,29,49,50]. Acute and repeated combined intracerebroventricular (i.c.v.) treatment with basic fibroblast growth factor (FGF2) and the 5-HT1AR agonist 8-OH-DPAT produced evidence of robust and highly significant antidepressant-like actions in the FST [6,13] due to synergistic allosteric receptor–receptor interactions. Increased recruitment of β-arrestin2 to the 5-HT1A protomer was observed together with an increased participation of 5-HT1A homodimers in the heteroreceptor complex upon combined FGF2 and 5-HT1AR agonist treatment [15,39].

### 2.1. Neurophysiological Studies

In a recent paper [11], it was found in control Sprague Dawley rats that FGF2 and a FGFR1 receptor agonist Sun-11602 diminished the currents over the G protein-coupled inwardly rectifying potassium channels (GIRK) produced by a 5-HT1A receptor agonist in pyramidal nerve cells of the CA1 region (Ammon's horn). An allosteric antagonistic receptor–receptor interaction may mediate these effects in the 5-HT1A–FGFR1 heteroreceptor complex. Previously, 5-HT1AR was shown to be located at the soma-dendritic level of the hippocampal pyramidal neurons [51]. Similar events may also take place in the 5-HT1A autoreceptor, known to be coupled to GIRK channels, and be part of the F5-HT1A–FGFR1 heteroreceptor complexes in the dorsal raphe [13,40,52,53]. However, such interactions remain to be analyzed.

### 2.2. Acute i.c.v. Effects of FGF2 and a 5-HT1A Agonist in a Genetic Rat Model of Depression Compared with Control Sprague Dawley (SD) Rats

#### 2.2.1. Behavioral Analysis

The Flinders sensitive line (FSL) rats were chosen, with the SD rat as a control. The FSL rats demonstrate a number of depression-like symptoms, for example, behavioral distress and deficiency in learning and memory [54]. I.c.v. injections of FGF2 and/or 8-OH-DPAT in the FSL and control strains were made and their acute effects over 48 hours evaluated in the forced swim test. The SD rats, as observed previously [6], demonstrated a reduction of the immobility time upon combined treatment with FGF2 and 8-OH-DPAT, not observed with single treatment. Such synergistic interactions were not observed in the genetic rat model of depression, which is of high interest [11]. Instead, in FSL rats, a reduction of immobility time was found after treatment with the 5-HT1A agonist treatment alone, which was blocked by combined treatment with FGF2. A neurophysiological correlation to these differential changes obtained in the FSL rats has not yet been obtained. However, a link to alterations in the 5-HT1A–FGFR1 heteroreceptor complexes does exist.

#### 2.2.2. In Situ PLA Analysis

In the control rats, it was of substantial interest to find that the combined, but not single treatment, selectively increased the number of 5-HT1A–FGFR1 heteroreceptor complexes in the CA2 area, but not in CA1 and CA3 areas [11]. The CA2 pyramidal cells have special features with projections especially to the deep layer of the CA1 pyramidal cells [55]. Furthermore, the CA2 projections strongly control the ventral CA1 efferents to the prefrontal cortex and the basolateral amygdala [56]. In addition, the CA2 projections appear to have a key role in social memory [57], which is in line with the current observations that the CA2 projections may have a role in depression.

Moving to 5-HT1A–FGFR1 heteroreceptor complexes of the FSL rats, single treatment with the 5-HT1A receptor agonist alone, but not combined treatment, significantly increased the number of 5-HT1A–FGFR1 heteroreceptor complexes in the CA2 and CA3 areas of the dorsal hippocampus [11]. These results match the behavioral data and indicate that 5-HT1A agonist treatment alone, by increasing the hippocampal 5-HT1A–FGFR1 heteroreceptor complexes in the CA2 and CA3 regions, can contribute to the antidepressant-like effects observed with 8-OH-DPAT alone. It may be proposed that the differential effects observed in control versus FSL rats can be related to differential composition and stoichiometry of the 5-HTR1A–FGFR1 receptor complexes in the control rats compared with the depressed FSL rats. The allosteric receptor–receptor interactions may therefore change, and a different pharmacology can then also develop.

Significant increases in the 5-HT1A–FGFR1 autoreceptor complexes were observed in the dorsal raphe after treatment with the 5-HT1A agonist in the absence or presence of FGF2 cotreatment in the control rat [11]. However, antidepressant-like effects were only noticed after combined i.c.v. treatment. It may therefore be that FGF2 treatment is necessary for the reduced coupling of the 5-HT autoreceptor to the GIRK channel in this autoreceptor complex. This will lead to reduced hyperpolarization and increased firing of the ascending 5-HT pathways originating from this nucleus and thus to antidepressant activity [58,59,60].

It should be noticed that in the FSL rats, no changes in the density of 5-HT1A–FGFR1 autoreceptor complexes were observed in the dorsal raphe of the FSL rat after 5-HT1A receptor agonist and/or FGF2 treatment. The reason for this is unclear, however, because there was an indication for a possible increase of the 5-HT1A–FGFR1 autoreceptor complexes in the dorsal raphe versus the control rat. Such an increase may counteract an additional increase in these 5-HT1A autoreceptor complexes upon treatment with 5-HT1A agonist and/or FGF2. Further studies are therefore necessary in combination with a neurophysiological analysis to evaluate if a compensatory reduction of 5-HT1A autoreceptor coupling to GIRK channels has developed in the FSL rats in the dorsal raphe to increase firing in the ascending 5-HT pathways.

The antidepressant-like effect found in the FSL rats after i.c.v. treatment with 5-HT1A receptor agonist alone may be produced by the ability of this treatment to increase the 5-HT1A–FGFR1 heteroreceptor complexes in the CA2–CA3 areas of the dorsal hippocampus [11]. In contrast, in control rats, only combined treatment produced increases in these heteroreceptor complexes and only in the CA2 areas of the dorsal hippocampus. It seems possible that the allosteric receptor–receptor interactions in these receptor complexes differ between treatments due to their differences in composition and stoichiometry in the two rat strains. As a result, the densities may vary with the pharmacological treatment as well as the allosteric modulation of the 5-HT1A coupling to the GIRK channels, with alterations in the firing of the pyramidal nerve cells of the CA2 and CA3 areas. 

## 3. 5-HT1A–5-HT2A Isoreceptor Complexes

There exist 5-HT1A–5-HT7 isoreceptor complexes, as demonstrated in cellular models [61]. They appear to be in equilibrium with 5-HT1A and 5-HT7 homodimers and with monomers. The allosteric receptor–receptor interaction is characterized by the ability of 5-HT7 to inhibit the Gi/o-mediated 5-HT1A signaling, leading to a reduction of the ability of 5-HT1A receptors to open the GIRK channels [61].

According to the triplet puzzle theory [62], triplet amino acid homologies participate in the receptor interface and guide the two receptors towards each other. This was true also for the 5-HT1A–5-HT2A isoreceptor complex [34], in view of the fact that 5-HT1A and 5-HT2A receptors also possessed two triplet amino acid homologies in a putative interface. The existence of a 5-HT1A–5-HT2A isoreceptor complex was therefore postulated [34] (Figure 1).

Evidence for its existence was obtained using BRET (Bioluminescence Resonance Energy Transfer Method) and in situ proximity ligation assay (in situ PLA) in cellular models. The existence of the 5-HT1A–5-HT2A isoreceptor complex in the brain was also found with in situ PLA. The complex was present in the pyramidal cell layer of the CA1–CA3 areas and in the anterior cingulate cortex [34]. The complexes in the hippocampal regions were studied 24 hours following the forced swim test and found to be vulnerable to the stress, shown by their marked reduction in the CA1–CA2 areas. As to the allosteric receptor–receptor interactions, antagonistic 5-HT2A–5-HT1A interactions were demonstrated both in the hippocampus and in the frontal lobe. 

A standard 5-HT2A agonist reduced the affinity of the 5-HT1A agonist binding sites in the two regions. Postjunctional 5-HT1A receptors in the forebrain are regarded as partly mediating antidepressant effects of selective serotonin reuptake inhibitors [23,26]. Therefore, a dominance of 5-HT2A receptor protomer activity may contribute to depressive effects in mood disorder through this mechanism. In line with these results, it was indicated early on that classical antidepressant drugs may block 5-HT2A receptors [63,64]. Depressed patients show a higher density of 5-HT2A receptors than normal patients, indicating a role of 5-HT2A receptors in depression development [65]. Thus, these two major 5-HT receptor subtypes interact in heteroreceptor complexes in limbic regions, which can contribute to their role in depression.

## 4. Multiple GalR–5-HT1A Heteroreceptor Complexes: Focus on GalR1–GalR2 Heterodimer and GalR1–GalR2–5-HT1A Heteroreceptor Complexes

### 4.1. Galanin N-Terminal Fragment (Gal (1–15))

The receptor for the galanin N-terminal fragment (Gal (1–15)) is the GalR1–GalR2 isoreceptor dimer [38,66,67], for which Gal (1–15) has a high affinity. It is of high interest that Gal (1–15) peptide given alone exerts strong depression-related and anxiogenic-like effects by targeting GalR1–GalR2 heterocomplexes in the raphe-limbic 5-HT system [67]. In contrast, Gal (1–15) enhances the antidepressant actions of the 5-HT1A receptor agonist 8-OH-DPAT [42]. The putative existence of a trimeric GalR1–GalR2–5-HT1A heteroreceptor complex was therefore postulated. The existence of the trimeric receptor complex may lead to novel allosteric receptor–receptor interactions which can help explain this interesting enhancement by Gal (1–15) of the antidepressant effects of 8-OH-DPAT [42].

This rather marked switch in the action of Gal (1–15) from inducing depression into antidepressant activity when coactivated with the 5-HT1A receptor agonist may be related to the Gal (1–15)-induced increases in the Bmax values of 5-HT1A high-affinity agonist binding sites. This takes place in the CA1 area and the dentate gyrus of the hippocampus, while a small reduction in the dorsal raphe develops where the 5-HT1A autoreceptors are located [42]. These results were matched by a Gal (1–15)-induced increase in the 5-HT1A mRNA levels in the hippocampus and a reduction of these levels in the dorsal raphe. Such changes may help increase the signaling over the 5-HT1A receptor in the hippocampus and reduce its signaling over the 5-HT1A autoreceptor. 

Nevertheless, this increase in the density of hippocampal 5-HT1A receptors takes place in the presence of a Gal (1–15)-induced reduction in the affinity of the high-affinity hippocampal 5-HT1A agonist binding sites [42]. Thus, the increase in 5-HT1A-mediated 5-HT neurotransmission may mainly develop with increased extracellular 5-HT levels, which may develop because the 5-HT autoreceptor levels appear to become reduced. Further work is needed to understand the mechanism for the recently discovered and highly interesting ability of Gal (1–15) to enhance the antidepressant actions of the 5-HT1A receptor agonist. It is clear, however, that 5-HT1AR can be in close proximity to both GalR1 and GalR2 using in situ PLA. Galanin appears to mainly target the GalR1–5-HT1A and GalR2–5-HT1A heteroreceptor complexes, while Gal (1–15) mainly targets the GalR1–GalR2–5-HT1A complex. 

**Figure 2:**
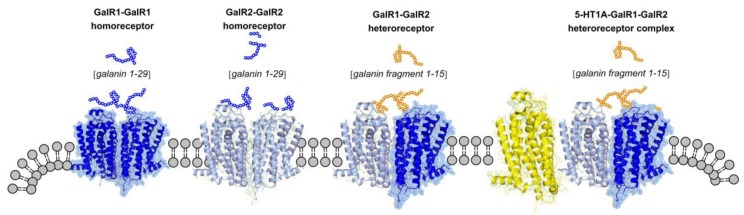
Illustration of the GalR1 and GalR2 homoreceptor complexes and the GalR1–GalR2 and 5-HT1A–GalR1–GalR2 heteroreceptor complexes in the plasma membrane. The major endogenous ligand for GalR1 and GalR2 homodimers is galanin 1–29, while the major ligand for the GalR1–GalR2 heterodimer is galanin 1–15. In the heterotrimer 5-HT1AR–GalR1–GalR2, the galanin fragment 1–15 may enhance the signaling of the 5-HT1A receptor protomer via a facilitatory allosteric receptor–receptor interaction involving mainly GalR2–5-HT1A receptor–receptor interactions.

It was proposed that in the GalR1–GalR2–5-HT1A heteroreceptor complex, the action of GalR2 is altered from enhancing Gi/o-mediated signaling of GalR1 in the GalR1–GalR2 heterodimer [66] into enhancing 5-HT1A receptor signaling in the trimer complex by, inter alia, reducing 5-HT1AR internalization from the plasma membrane [42]. In addition, GalR2 may also maintain its signaling over Gq and PLC. In line with these results, it was found that Gal (1–15) enhanced the antidepressant effects of the SSRI fluoxetine using the forced swim test [43] (Figure 2). In this case, however, extracellular 5-HT levels were elevated, likely increasing 5-HT signaling not only over the 5-HT1A receptor, but also over a number of other 5-HT receptor subtypes known to produce antidepressant actions. As an example, the 5-HT4R can be mentioned, a fast-onset antidepressant [26,29,68,69].

In the combined treatment with Gal (1–15) and fluoxetine, the modulation of the 5-HT1A agonist binding sites in the dentate gyrus was also different from the modulation found with Gal (1–15). The modulation by combined treatment with fluoxetine and Gal (1–15) produced an increased affinity of the 5-HT1A agonist binding sites and a reduction of their Bmax values in the dentate gyrus [43], in contrast to the reduced affinity and increased Bmax levels found in this region with Gal (1–15) [42]. However, the increase in 5-HT1A mRNA levels in the hippocampus was still obtained after the combined treatment with Gal (1–15) and fluoxetine, as found after treatment with Gal (1–15) alone. It was suggested based on these observations that Gal (1–15) given intranasally in depression may offer a new treatment when combined with SSRIs or 5-HT1A receptor agonists to improve their antidepressant actions. The impact of the 5-HT1A receptors for the antidepressant actions was indicated from the observations that a 5-HT1A receptor antagonist counteracted the antidepressant effects [11].

### 4.2. Galanin (Gal (1–19))

As for galanin, which is a 29-amino acid neuropeptide (Gal (1–29)), it binds with high affinity to GalR1, GalR2, or GalR3 receptors and shows a reduced affinity for the GalR1–GalR2 heteroreceptor complexes [38,66] (Figure 2). It was proposed that galanin can contribute to depression by inhibition of firing in the dorsal raphe 5-HT nerve cells, sending projections into the telencephalon and diencephalon [44,70]. In the FSL rat model of depression, an increase in the galanin receptor binding sites was found in the dorsal raphe, giving support to this view [71].

GalR1–5-HT1A heteroreceptor complexes were also demonstrated [37,46], and later on, also GalR2–5-HT1A heteroreceptor complexes [42] (Figure 1). GalR2 is known to have antidepressant activity, in contrast to GalR1, which can contribute to depressive actions. This research illustrates how galanin and its fragment Gal (1–15) can produce differential changes in mood from depressive to antidepressant actions, being dependent on how the galanin receptor subtypes come together with the 5-HT1A receptors and probably other types of 5-HT receptor subtypes. The heteroreceptor complexes formed from the GalR and 5-HT1A receptor subtypes with various stoichiometries will determine their functions and role in major depression. The molecular complexes formed are likely highly dynamic and vary from one brain region to another one. The hippocampus appears to be a major target.

## 5. Isodimers and Heterodimers on the Receptor Interface, Especially of Serotonin Receptor Homodimers

In the serotonin homo-, iso-, and heteroreceptor complexes, the allosteric communication between the involved protomers takes place via the receptor interface. Thus, the interface interaction becomes a key player and the understanding of the receptor interface necessary. Therefore, there is a significant body of experimental and bioinformatic work that has focused on identifying key residues of the receptor interface for serotonin homo- and heteroreceptor complex formation. 

Based on biophysical and biochemical techniques, for instance, FRET (fluorescence resonance energy transfer), BRET, coimmunoprecipitation, and mass spectrometry, several models of serotonin receptor dimerization have been proposed. Some receptor domains have also been identified to be involved in this phenomenon. The transmembrane (TM) domain disulphide bonds (Cys112 and Cys145) play a key role in the homodimerization of 5-HT4R [72]. Instead, a negatively charged motif at the C-terminal tail of 5-HT2AR drives the heterodimerization of 5-HT2AR with the D2R by means of the formation of noncovalent complexes [73]. This high-energy strength of double arginine-phosphate electrostatic interaction interface mechanisms has also been found in the receptor interface of the A2AR–D2R heteromer. It possesses a covalent-like stability, as demonstrated with mass spectrometry and site-directed mutagenesis and BRET techniques [74,75]. On the other hand, TM helix domains (especially the TM4 and TM5) are an important part of the receptor interface in the 5-HT1A [76] and 5-HT2C [77] homoreceptor complexes. The participation of TM4 and TM5 in the oligomer interface has also been demonstrated within other complexes such as the serotonin 5-HT4 receptor homodimer [72], the chemokine CCR5 receptor homodimer [78], the α-factor pheromone receptor (Ste2) homodimer [79], the corticotrophin-releasing hormone receptor/arginine vasotocin receptor heterodimer [80], and the serotonin 5-HT2A/metabotropic glutamate receptor 2 heterodimer [81]. Furthermore, the transmembrane helixes (TM-5) and (TM-8) of 5-HT1A were proposed to participate in the interface of the 5-HT1A–FGFR1 heteroreceptor complexes [6,40]. Overall, today it is well-accepted and demonstrated that transmembrane helices participate in class A, B, and C GPCR homo- and heterodimerization. The interaction interfaces are formed by lipid-exposed surfaces within the transmembrane helical bundle of each individual protomer. Thus, alterations in the receptor hydrophobic core, particularly residues containing the ligand-binding site, predictably affect the receptor conformation and oligomerization. 

Also based on mathematical and bioinformatic approaches, it is possible to understand the receptor–receptor interface interaction. For instance, it was proposed that structurally homologous amino acid residues can play a role in the receptor interface [62]. Through mathematical models and bioinformatic analysis, it became possible to demonstrate that known heterodimers, but not nonheterodimers, contained a number of protriplets in a putative receptor interface [62,82]. These protriplets appeared to be essential for the coming together of the two receptors into a heterodimer. The protriplet puzzle theory was introduced in 2010, but contratriplets may also exist [62,83]. Nonintersecting sets of protriplets and contratriplets were obtained from various lists of receptor pairs [82]. Any protriplet is shown to be a homology in at least one heterodimer, but is not found as a homology in any nonheterodimer, and promotes heteromerization. The protriplets theory makes sense because hot spots in the receptor interface are often protected by residues that are not relevant for binding, but the role of which is to isolate the hot spots from the surrounding solvent [82,84]. 

It should be noticed that protriplet amino acid homologies have been identified in all the serotonin iso- and heteroreceptor complexes in the current article (Table 1). In the 5-HT1A–FGFR1 heteroreceptor complex, the TLG (Thr–Leu–Gly) and AAR (Ala–Ala–Arg) protriplets were demonstrated [6,34]. In the 5-HT1A–5-HT2A isoreceptor complex, the LLG (Leu–Leu–Gly) and QNA (Gln–Asn–Ala) protriplets were found [34]. In the 5-HT1A–GalR1 and 5-HT1A–GalR2 heteroreceptor complex, protriplets LLG, LAR (Leu–Ala–Arg), and RNA (Arg–Asn–Ala) were identified, which was true also for the GalR1–GalR2 isoreceptor complex [34,37].

The findings that the different types of GalR isoreceptor and heteroreceptor complexes use the same protriplets, leading to competition for the same receptor interface, can help explain the alteration in the allosteric receptor–receptor interactions found in the putative GalR1–GalR2–5-HT1A heteroreceptor complex. As postulated, it may be that in the trimeric complex, the GalR2 signaling dominates, including an enhancement of 5-HT1A signaling through a positive allosteric mechanism.

## 6. Concluding Remarks

**Figure 3:**
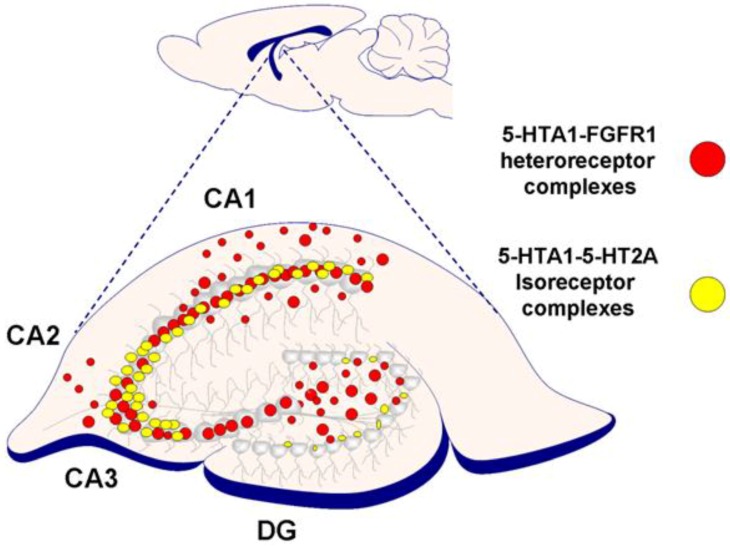
Illustration of the distribution pattern of 5-HT1A–FGFR1 heteroreceptor and 5-HT1A–5-HT2A isoreceptor complexes visualized with PLA in the rat dorsal hippocampus. The 5-HT1A–FGFR1 heteroreceptor complexes, in red, have a more widespread distribution, mainly located within the pyramidal cell layer of the CA1–CA4 and in the plexiform cell layer of the dentate gyrus. However, they exist in low densities in the stratum oriens and radiatum of the CA1–CA4 regions and the subgranular layer. Instead, the 5-HT1A–5-HT2A isoreceptor complexes, shown in yellow, are concentrated to the pyramidal cell layer of the CA1–CA3 regions, with some located in the subgranular layer of the dentate gyrus.

It appears clear that multiple 5-HT1A heteroreceptor complexes exist in the hippocampus, modulating its GABA and glutamate synapses. Through this modulation, these heteroreceptor complexes change the activity of the hippocampal networks and thus their outputs to mood-regulating limbic areas. Their modulation of these networks may mediate the antidepressant actions produced by 5-HT1A receptor activation. It is not known which of the 5-HT1A heteroreceptor complexes plays a leading role in reducing depression: 5-HT1A–FGFR1, 5-HT1A–5-HT2A, GalR–5-HT1A, or GalR1–GalR2–5-HT1A heteroreceptor complexes. It remains to establish if these different 5-HT1A complexes can modulate the same or different glutamate and GABA synapses in the hippocampus. Their architecture in terms of their individual distribution in the CA1–CA4 regions and the dentate gyrus and their layers is still relatively unknown and should be determined. They mainly have a postjunctional location. As an example, it is shown in Figure 3 that the 5-HTA–5-HT2A isoreceptor complexes are mainly located in the pyramidal cell layer of CA1–CA3 areas, with few complexes in other regions of the dorsal hippocampus. It will be of high interest to determine if some of them are in balance with each other, which can be altered by 5-HT and other transmitters. Future work should determine which complex is the most vulnerable in depression development. Restoring its activity may be of special value to reducing depression.

## Figures and Tables

**Table 1:** Demonstration of protriplet amino acid homologies in 5-HT1AR–FGFR1, 5-HT1AR–5-HT2AR, and GalR1–GalR2 heterodimers.

	Galanin Set					Serotonin Set			2010 [37]		2012 [6]	2017 [34]			
	VLA	NGS	GAF	LIF	LAA	SLA	VLV	DVL	LAR	SNS	AAR	LLG	TLG	QNA	RNA
5-HT1AR			+	+		+	+	+	+	+	+	+	+	+	+
FGFR1	+	+	+		+		+		+		+	+	+		
GalR1	+	+	+	+	+		+		+	+		+			+
GalR2	+	+		+	+		+								+
5-HT2AR						+		+				+	+	+	

Some protriplets were reported in in previous works by Borroto-Escuela et al. [6,34,37]. Others were found in lists (sets) of receptor pairs mainly involving galanin or serotonin receptors. The protriplets only exist in heterodimers and are not found in nonheterodimers. At least the majority of these protriplet homologies appear to be part of the receptor interface. (+)it refer to the presence of the protriplet in the protomer.

## Abbreviations

The following abbreviations are used in this manuscript:

5-HT1A	serotonin 5-HT1A receptor subtype
5-HT2A	serotonin 5-HT2A receptor subtype
CNS	central nervous system
FGFR1	fibroblast growth factor receptor 1 subtype
FSL	Flinders sensitive line rats
FST	forced swim test
Gal (1–15)	galanin N-terminal fragment peptide (1–15)
Gal (1–29)	galanin peptide (1–29)
GIRK	G protein-coupled inwardly rectifying potassium channels
GPCR	G protein-coupled receptor
i.c.v.	intracerebroventricular
RTK	receptor tyrosine kinase
SD	Sprague Dawley rats
